# Potential contribution of intrinsic developmental stability toward body plan conservation

**DOI:** 10.1186/s12915-022-01276-5

**Published:** 2022-04-11

**Authors:** Yui Uchida, Shuji Shigenobu, Hiroyuki Takeda, Chikara Furusawa, Naoki Irie

**Affiliations:** 1 Center for Biosystems Dynamics Research, RIKEN, 6-2-3 Furuedai, Suita, Osaka, 565-0874 Japan; 2 NIBB Core Research Facilities, National Institute for Basic Biology, Okazaki, 444-8585 Japan; 3 Department of Biological Sciences, Graduate School of Science, The University of Tokyo, Tokyo, 113-0033 Japan; 4 Universal Biology Institute, The University of Tokyo, 7-3-1 Hongo, Bunkyo-ku, Tokyo, 113-0033 Japan

**Keywords:** Evolution, Phylotypic period, Developmental hourglass model, Developmental stability, Canalization, Robustness, Transcriptome

## Abstract

**Background:**

Despite the morphological diversity of animals, their basic anatomical patterns—the body plans in each animal phylum—have remained highly conserved over hundreds of millions of evolutionary years. This is attributed to conservation of the body plan-establishing developmental period (the phylotypic period) in each lineage. However, the evolutionary mechanism behind this phylotypic period conservation remains under debate. A variety of hypotheses based on the concept of modern synthesis have been proposed, such as negative selection in the phylotypic period through its vulnerability to embryonic lethality. Here we tested a new hypothesis that the phylotypic period is developmentally stable; it has less potential to produce phenotypic variations than the other stages, and this has most likely led to the evolutionary conservation of body plans.

**Results:**

By analyzing the embryos of inbred Japanese medaka embryos raised under the same laboratory conditions and measuring the whole embryonic transcriptome as a phenotype, we found that the phylotypic period has greater developmental stability than other stages. Comparison of phenotypic differences between two wild medaka populations indicated that the phylotypic period and its genes in this period remained less variational, even after environmental and mutational modifications accumulated during intraspecies evolution. Genes with stable expression levels were enriched with those involved in cell-cell signalling and morphological specification such as *Wnt* and *Hox*, implying possible involvement in body plan development of these genes.

**Conclusions:**

This study demonstrated the correspondence between the developmental stage with low potential to produce phenotypic variations and that with low diversity in micro- and macroevolution, namely the phylotypic period. Whereas modern synthesis explains evolution as a process of shaping of phenotypic variations caused by mutations, our results highlight the possibility that phenotypic variations are readily limited by the intrinsic nature of organisms, namely developmental stability, thus biasing evolutionary outcomes.

**Supplementary Information:**

The online version contains supplementary material available at 10.1186/s12915-022-01276-5.

## Background

Phenotypes of animals are not freely changeable during evolution. For example, basic anatomical features of animals in the same phylum, or the body plan, have exhibited striking conservation throughout hundreds of millions of years of evolution [1]; this is often regarded as a typical example of phylogenetic inertia [2, 3]. However, the factors driving this strict conservation remain a matter of debate. Recent findings have supported the classic idea [4, 5] that one of the main biasing factors for this conservation exists in embryogenesis itself. The body plan establishing phase—the phylotypic period [4–6]—has been a continuous target of conservation throughout vertebrate evolution [7–14]. In the meanwhile, few mechanisms have been proposed for the conservation of the phylotypic period. One idea was that the phylotypic period remained conserved due to the near absence of diversifying selective pressure, when compared to variable earlier and later stages which are susceptible to adaptive changes [15, 16]. Along with this, Liu et al. found more frequent signatures of positive selections on potential regulatory regions utilized in the earlier and later Drosophila embryonic stages than their mid-embryonic stages [17]; however, whether the positive selection per se is sufficient for the hourglass-like divergence remained to be clarified. With this regard, Zalts et al. tested this by experimental evolution of nematodes under near absence of positive selection [18] and demonstrated that mid-embryonic phase show highly conserved among the experimental lines established under this condition. This indicates that absence of positive selection per se may not be sufficient to explain the conservation of the phylotypic period. Indeed, considering the rapid accumulation of mutations by neutral evolution, it is reasonable, at least theoretically, to assume that the absence of positive selection has much less to do with retainment of phenotype through hundreds of millions of evolutionary years. Alternatively, another contrasting idea was that relatively strong negative (purifying) selection acted on the phylotypic period through embryonic lethality [5, 19]. We previously tested this using three vertebrate species and found that not only lethality rate under the development of healthy condition, but also under harsh conditions such as development after ultraviolet (UV) irradiation up to lethal dose 50 (LD50), treatment of transcriptional inhibitors, nor translational inhibitors did not lead to higher lethality rate in the phylotypic period [20]. These suggest that neither near absence of diversifying selections, nor strong negative selection through embryonic lethality to the phylotypic period is not sufficient to explain its evolutionary conservation.

Alternatively, an attractive but untested scenario is that the phylotypic period has limited potential for generating phenotypic variations; the lack of new phenotypic variations would decelerate diversification, even under diversifying positive selection. In accordance with this idea, our previous study suggested that the phylotypic period buffers mutational or environmental perturbations, or both, to create phenotypic variations [20], suggesting its highly canalized [21, 22] status. Consistent with this idea, studies using Drosophila reported that the potential phylotypic period in arthropods showed a regulatory feature of robustness in gene expression patterns against stochastic noise [23]. A theoretical study [24] further predicted that this canalized status is an emergent property of developmental stability, a feature by which the same phenotype is shown under developmental noise but without mutational and environmental perturbations [25]. In other words, under this prediction, stable phenotypes which show fewer stochastic phenotypic variations not only leads canalized status, but also to their evolutionary conservation [26, 27]. However, this scenario awaits empirical verification. Here, by using both highly homogeneous inbred and naturally diversified wild medaka populations, we tested this hypothesis by estimating canalized status, developmental stability, and their relationship to evolutionary conservation. Since the phenotypic conservation of the phylotypic period has been verified by comparing whole embryonic transcriptomes, with an assumption that it reflects similar composition of cell types, we also followed this strategy. Specifically, by measuring the whole embryonic transcriptome as the embryo phenotype, we evaluated canalized status as a reduction in the abundance of variations against the mutations and environmental changes experienced during intraspecies evolution of populations. Developmental stability was estimated as a reduction in variable gene expression in inbred twins of the same gender.

## Results

**Fig. 1:**
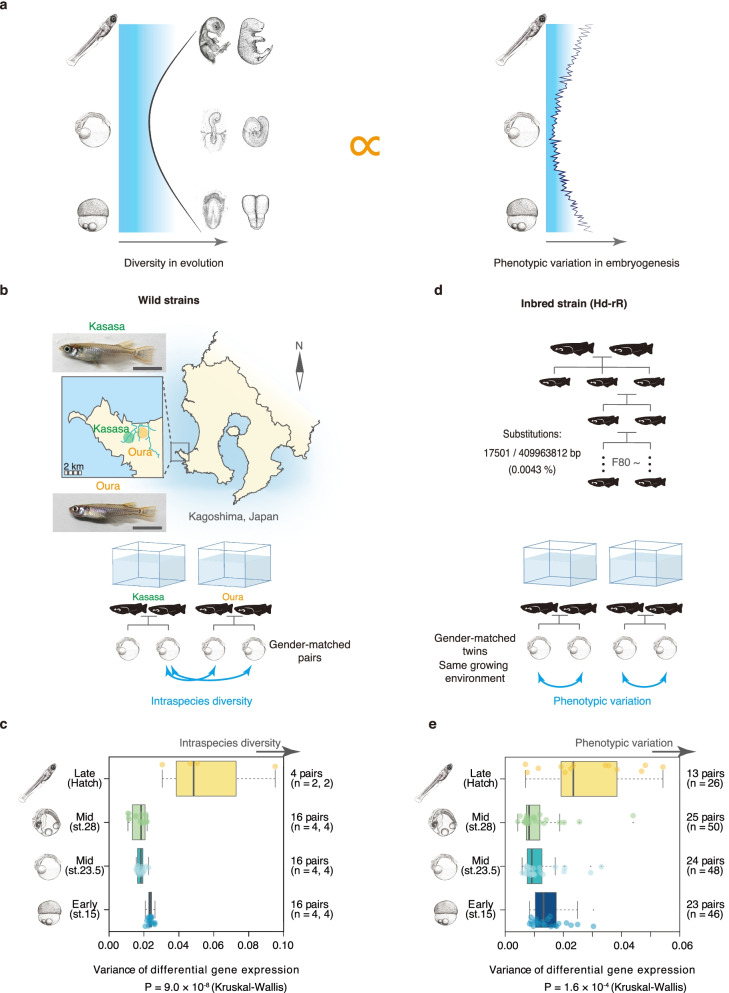
Correspondence between evolutionary conservation and phenotypic variation during embryogenesis in medaka embryos. **a** Schematic representation of the relationship between developmental stability and evolutionary conservation of the body plan establishment period in vertebrates [4, 5, 7, 10–12]. This hypothesizes that phenotypic variation in the absence of genetic diversity (right) is correlated with evolutionary diversity (left). **b** Wild medaka populations (Kasasa and Oura) used for measuring microevolutionary conservation. Whole embryonic transcriptomes were compared between gender-matched embryos from each population raised in the same environment. **c** Microevolutionary conservation evaluated in four developmental stages. Variance of distribution of differential gene expression levels was used to represent intraspecies phenotypic differences (See also ‘Methods’). **d** An inbred medaka strain (Hd-rR) was used to estimate phenotypic variations. Whole embryonic transcriptomes of gender-matched twins raised in the same environment were compared. **e** Whole embryonic phenotypic variations were quantified by the variance of distribution of differential gene expression levels (See also ‘Methods’). The Kruskal–Wallis test (*P* value shown) followed by multiple comparisons (Steel–Dwass) indicated that st. 23.5 and st. 28 had significantly smaller phenotypic variation and intraspecies diversity than the earlier and later stages (st. 15 vs. st. 23.5, *P* = 8.4 × 10^–3^; st. 15 vs. st. 28, *P* = 1.1 × 10^–3^; st. 15 vs. Hatch, *P* = 1.3 × 10^–2^; st. 23.5 vs. st. 28, *P* = 0.91; st. 23.5 vs. Hatch, *P* = 1.3 × 10^−2^, st. 28 vs. Hatch, *P* = 1.3 × 10^−2^) and significantly smaller phenotypic variation (st. 15 vs. st. 23.5, *P* = 4.7 × 10^−2^; st. 15 vs. st. 28, *P* = 1.7 × 10^−2^; st. 15 vs. Hatch, *P* = 5.7 × 10^−2^; st. 23.5 vs. st. 28, *P* = 0.79; st. 23.5 vs. Hatch, *P* = 1.1 × 10^−2^; st. 28 vs. Hatch, *P* = 5.8 × 10^−3^). Box plots: centre line, median; box limits, upper and lower quartiles; whiskers, 1.5× interquartile range; points, outliers (**d, e**)

Our previous study indicated that phylotypic periods of vertebrates are robust against extreme mutational and perturbations under laboratory conditions [20], implying their canalized status. We tested here whether the phylotypic period of medaka [7, 10, 12, 28] also had canalized status against mutations accumulated under natural conditions. For this purpose, we used two wild populations of medaka (*Oryzias latipes*), namely the Kasasa and Oura populations, which reside in the same river system within 5 km apart from each other. Gene expression profiles obtained from individual embryos were compared as multivariate phenotypes [10, 14] (Fig. 1a,b). As expected, the results indicated that the smallest inter-individual variations between the two wild populations were found at stage (st.) 23.5 and st. 28, in the previously identified phylotypic period in medaka [7, 10, 12, 28] (Fig. 1c). This canalized trend was also corroborated by comparisons between more genetically distantly related medaka populations (Hd-rR vs. Kasasa, *P* = 3.3 × 10^–50^; Hd-rR vs. Oura, *P* = 9.7 × 10^–53^; Kruskal–Wallis tests; Additional file 1: Figure S1). These results not only indicate that the phylotypic period remained conserved during the diversification of medaka populations, but it also implies that the phylotypic period has limited potential for creating phenotypic variations, even with the genetic mutations accumulated during the intraspecies period of evolution.

However, these findings do not necessarily indicate that the phylotypic period has less potential to produce phenotypic variations than other stages, because the mutations accumulated during the diversification of these medaka populations could have contributed to phenotypic diversification at earlier and later stages. We therefore set out to determine whether the phylotypic period has developmental stability—in other words, whether there are relatively few phenotypic variations, even in the absence of mutational effects, but in the presence of the stochastic noises that arise during development.

As an approximation of developmental stability, we carefully measured variations in the whole embryonic transcriptome between gender-matched twins of a highly inbred medaka line (Hd-rR) raised in exactly the same environment (13 to 25 pairs for each developmental stages). This comparison between twins, rather than comparison among multiple embryos within a population, enables us to measure developmental stability with minimal biases from unwanted factors, such as standing genetic variations, differences in parental and environmental conditions. Although the inbred medaka still had minor genetic differences among individuals, we confirmed that the nucleotide substitution rate between the gender-matched twins was much smaller (0.0043%) than those in the wild types (0.1%, Fig. 1d, Additional file 1: Figure S1). To reduce unwanted bias and noise when measuring transcriptomic variations by distance index between the gender-matched twins, we (i) adjusted the RNAseq read depths to avoid bias against expression levels (Additional file 1: Figure S2a, b); (ii) confirmed that the resolution of the distance index was high enough to classify different samples (Additional file 1: Figure S2c-e); and (iii) further controlled for technical errors by adopting only those genes that showed significantly larger deviations in inbred-twin samples than in technical replicates (Additional file 1: Figure S3).

After careful measurement of the phenotypic variation between the inbred twins, we found that the phylotypic period (st. 23.5, st. 28) [7, 10, 12, 28] had significantly smaller phenotypic variation than the earlier and later stages (Fig. 1e), indicating that it had greater developmental stability against stochastic noises in the phylotypic period with minimal mutational effects. This trend of stability was consistently observed in analyses with male- and female-specific embryos, with genes associated with the developmental process, and with constitutively expressed genes (Additional file 1: Figure S4a-c). A similar trend was observed for all five categories of genes grouped by expression level (Additional file 1: Figure S4d). These results, together with the robust, canalized nature of the phylotypic period against extreme mutational or environmental perturbation [20], suggest that the phylotypic period have high stability and canalized status, which may have led to its evolutionary conservation through lower potential to produce phenotypic variations than other stages.

To test whether the developmental stability of the phylotypic period arose from the cumulative effects of stability in individual genes, we next evaluated the stability of each gene expression level by using gender-matched inbred twins (Additional file 1: Figure S5a). As expected, there was enrichment of genes expressed at stable levels in the phylotypic period (st. 23.5 and st. 28) [7, 10, 12, 28]. A similar result was obtained, with intraspecies conservation of expression levels, between wild strains (Additional file 1: Figure S5b). However, this result has to be interpreted cautiously, as we did not control for a major factor potentially confounding both stability and conservation, namely, expression level [29–33]. To correct for this potential confounding factor, we used a running median correction [31], given that the expression levels of the genes were non-linearly correlated with their stability and conservation (Additional file 1: Figure S5c). In short, the median stability value was calculated for genes with similar expression levels (basically by using a sliding 501-gene window) and used to correct the potential bias of expression level toward stability (Additional file 1: Figure S5d, e; see also ‘Methods’) [31].

**Fig. 2:**
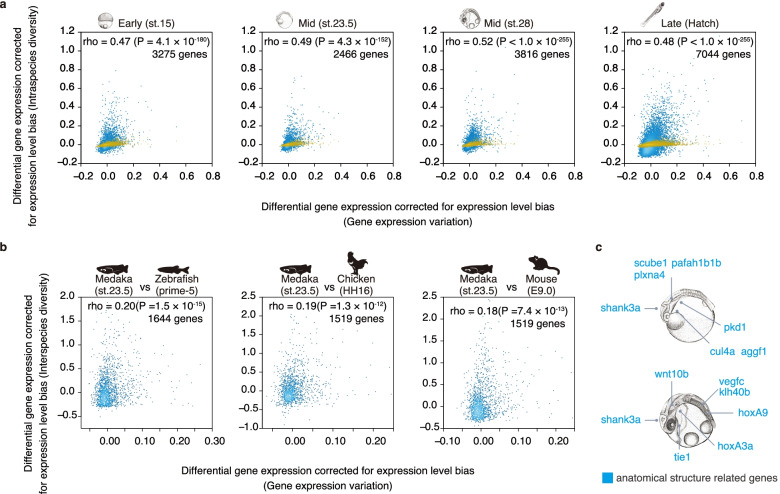
Genes with low variation between inbred twins tend to have evolutionarily conserved expression levels. **a** Scatter plots in light blue represent the relationship between variation in gene expression levels (*x* axis, differential expression levels between inbred twins) and intraspecies diversity (*y* axis, expression level difference calculated from Kasasa and Oura medaka embryos). Overlaid scatter plots in yellow represent the relationship between gene expression variation (*x* axis) and the technical error determined from technical replicates (*y* axis). **b** Relationships between gene expression variation as a reflection of stability in expression levels (*x* axis) and interspecies diversity (*y* axis) are shown for the macroevolutionarily conserved developmental period (st. 23.5) [7, 10, 12, 28] (left, medaka vs. zebrafish; centre, medaka vs. chicken; right, medaka vs. mouse). Spearman’s correlation coefficient between each variation of expression level is shown in each plot. *P* values are given for the test of no correlation. **c** Among the 10% of genes with the smallest variation detected at st. 23.5 and st. 28, representative genes associated with anatomical pattern development (GO:0048856) and anatomical structure formation involved in morphogenesis (GO:0048646) are shown. Tissues of expressed regions were referenced or deduced from studies in related vertebrates [36].

Consistent with pioneering studies based on unicellular organisms [34, 35], our results indicated that genes with smaller expression variation (calculated between the gender-matched inbred twins) tended to show more conserved expression levels at the microevolutionary scale in multicellular organisms (Fig. 2a, Hd-rR vs. Kasasa, Spearman’s rho = 0.62–0.78, *P* < 1.1 × 10^–150^; Hd-rR vs. Oura, Spearman’s rho = 0.61–0.80, *P* < 1.2 × 10^–145^; test of no correlation). Importantly, we confirmed that this tendency could not be explained by the noises arising from technical errors (Fig. 2a, yellow dots). What is more, weak but significant correlations (Spearman’s rho around 0.18 to 2.0) between stability and conservation were detected at the macroevolutionary scale. Specifically, by comparing 1:1 orthologs expressed in the phylotypic periods of other vertebrates (zebrafish, chickens, and mice) and medaka, we found that genes with low expression-level variation in medaka tended to show conserved expression levels in the species used for comparison (Fig. 2b). Of note, the genes with the least expression-level variation included those known to be involved in morphological patterning, such as *Hox* and *Wnt* genes (Fig. 2c, Additional file 1: Figure S6). These results suggest that developmentally stable genes tend to have conserved expression levels and may have contributed to conservation of the phylotypic period.

**Fig. 3:**
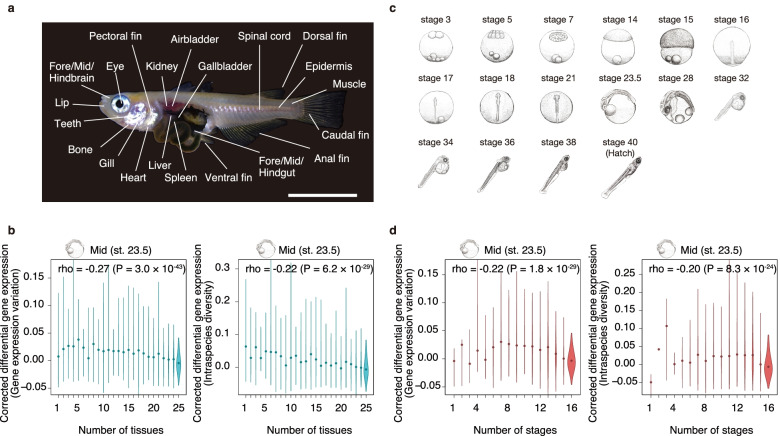
Genes with low expression variability and higher conservation tend to have high pleiotropy of expression. **a** Twenty-five kinds of tissues were each collected from each single adult medaka to avoid overlapping sampling and subjected to RNAseq to analyse the spatial pleiotropy of gene expression. Four adults were used for each tissue for this analysis as biological replicates. **b** The relationship between the number of expressed (mean TPM ≥ 1 among the four replicates) tissues and gene expression variation measured at st. 23.5 (left) is shown as a violin plot (left). The relationship between gene expression variation in the inbred twins (detected at st. 23.5) and intraspecies diversity is shown in the right panel. See also Additional file 1: Figure S7 for results obtained for the other stages. **c** Sixteen medaka developmental stages were used to analyse the relationship between expression variation and temporal pleiotropy. Previously published transcriptome data [39] (data on three biological replicates for each stage) were utilized. **d** The relationship between number of stages of expression (mean TPM ≥ 1 among the three replicates) and gene expression variation at st. 23.5 (left) is shown as a violin plot. A similar analysis against intraspecific diversity is shown in the right panel. In **b** and **d**, the violin plot represent genes within a 1.5× interquartile range, and the dot in the middle represents the median value. Spearman’s correlation coefficient and *P* values (test of no correlation) are shown in each plot

To look for a potential link between genes with stable expression levels and conservation of the phylotypic period, we next searched for a potential link with pleiotropic expression. Generally, genes expressed in a variety of biological processes (e.g. tissues or developmental stages)—pleiotropically expressed genes—tend to be evolutionarily conserved [37, 38]. Moreover, our recent study demonstrated that the phylotypic period is enriched with pleiotropically expressed genes [7], and this has potentially led to the period’s conservation through pleiotropic constraints [37, 38]. Accordingly, we tested whether genes with stable expression levels tended to show both spatial and temporal pleiotropic expression (Fig. 3). Spatial pleiotropy was estimated as the number of tissues in which the genes were expressed (RNAseq data of 25 adult medaka tissues were obtained for this), and temporal pleiotropy was estimated as the number of developmental stages in which the genes were expressed (RNAseq data of 16 developmental stages [39]). Consistent with previous studies, the expression levels of genes with spatial and temporal pleiotropic expression tended to be evolutionarily conserved (Fig. 3). Importantly, we also found a moderate but significant correlation between low expression variability and pleiotropic expression (Fig. 3 and Additional file 1: Figure S7). These results imply that *Cis*-regulatory factors could be a link among the pleiotropic, stable and conserved statuses of gene expression levels. However, no sign of this link was found in our further analyses. In brief, we analysed whether gene expression stability or microevolutionary conservation of gene expression was correlated with any features of the regulatory region [40] as determined by using assay for transposase accessible chromatin sequencing (ATAC-seq) data [28] (Additional file 1: Figure S8a-d); the mutation rate (Additional file 1: Figure S8e-g); or the TATA-box number [41] (Additional file 1: Figure S8h-j). The results showed that length [40], number, mutation rates, and TATA-box distributions [41] of potential regulatory regions do not show significant correlation with the stability of gene expression levels (Additional file 1: Figure S8).

Finally, we tested whether genes with stable expression expressed in st.23.5 and st.28 contributed not only to the conservation of the phylotypic period, but also to the development of the body plan. Our analysis indicated that several representative development-related genes were found among the 10% of genes with the least expression variation in st. 23.5 and st. 28 (Fig. 2c). Although Gene Ontology (GO) slim terms related to anatomical structure development (GO:0048646, GO:0048856) were not found to be significantly enriched among the 10% of genes with the least expression variation in the potential phylotypic period, those involved in cell–cell interactions (cell-cell signalling [GO:0007267], cell junction organization [GO: 0034330]) were found to be enriched more than 2 times compared with the remaining 90% of genes in st. 23.5 and st. 28 (Additional file 1: Figure S9). The GO-term-related genes included *Wnt10b*, *axin1*, *shank3a*, *trpm7*, and *gpc3* (Additional file 2: Table S1). *Wnt10* is known to be important for midbrain–hindbrain boundary formation [42], whereas *gpc3* is involved in regulation of hedgehog signalling and leads to a variety of phenotypes (including overgrowth and facial deformation) when mutated [43]. These results suggested that the genes with stable expression levels included those critically involved in body plan, albeit not as a whole.

## Discussion

Our results, together with those of previous studies [20, 23], indicate that the phylotypic period and its genes are not only stable against stochastic developmental noises (i.e. developmentally stable) but also robust against mutational and environmental perturbations (i.e. canalized). In addition, these stability and robustness of the phylotypic period and its genes correlated with intra- and interspecies level of evolutionary conservation, and genes with highly stable expression levels were enriched with genes involved in cell-cell communications. On the basis of the theoretical predictions that phenotypic stability correlates with evolutionary conservation [24, 26, 27], together with our results, we propose that the high developmental stability of the phylotypic period has contributed to the conservation of the body plan.

A limitation of our study is that we did not measure developmental stability in genetically identical individuals to completely exclude the effects of minor genetic differences. In addition, the mechanisms behind developmental stability and canalization remain largely unclear. The cost and careful controls of experimental conditions were also barriers for us to add further developmental stages. Future studies, such as experimental evolutionary studies using cloned embryos, with improved developmental time resolution would provide further solutions to the experimental design and answers to the evolutionary mechanism behind the developmental stability and canalization.

Extended modern synthesis explains the evolutionary process mainly in terms of the ways in which phenotypic variations backed by genetic mutations spread or disappear across generations. Our results highlight the concept that additional bias, toward phenotypic evolution, readily exists intrinsically in developmental systems. In the near future, this concept may pave the way for us to make quantitative predictions of phenotypic evolution on the basis of its stability.

## Conclusions

By analyzing single-embryonic transcriptomes of medaka lines, we found that the phylotypic period shows the highest developmental stability under minimal mutational and environmental effects, and this correlated with its evolutionary conservation in intraspecies level of evolution. The same correlational relationship between the stability and evolutionary conservation was also observed for each gene expression level. These results highlight a possibility that the body plan establishing, phylotypic period has less potential to create phenotypic variations along embryogenesis, and this may have contributed to limited phenotypic diversity of vertebrate body plan.

## Methods

### Animal care and embryo sampling

Experimental procedures and animal care were conducted in strict accordance with guidelines approved by the Animal Experiments Committee of the University of Tokyo (approval ID: AP19-8 and AP19-10). The inbred medaka strain (Hd-rR) was provided by NBRP (the National BioResource Project) at NIBB (the National Institute for Basic Biology, Aichi, Japan). Two wild strains, Kasasa (strain ID: WS1268) and Oura (strain ID: WS253), were supplied by NBRP at Utsunomiya University (Utsunomiya, Japan). These two strains are geographically close to each other and inhabit the same river system. They belong to the same sub-species group (southern population) as the inbred line Hd-rR [44]. The other two wild strains, Kaishi (strain ID: WS1275) and Tango (strain ID: WS240), were obtained from NBRP at Niigata University (Niigata, Japan). They belong to the southern population in Japan but inhabit different river systems. All adult medaka were maintained at 28 °C under a 14-h light:10-h dark cycle. Fertilized eggs were obtained by natural mating and incubated in hatching buffer at the same temperature used for keeping adults (28 °C). The embryos were carefully staged according to the standard developmental table [45]. The number of somites was used as a specific criterion to identify the stages of the phylotypic period (st. 23.5, fourteen somites; st. 28, thirty somites).

### RNA extraction and RNA sequencing

#### Embryos

After the identification of developmental stages, each embryo was homogenized in QIAzol reagent (Qiagen, Germantown, MD, USA), and total RNA was isolated by using an RNeasy Min Elute kit (Qiagen) in accordance with the standard protocol. Total RNA concentrations were quantified by using a Qubit 2.0 Fluorometer (Thermo Fisher Scientific, Waltham, MA, USA) with a Qubit RNA HS Assay Kit (Thermo Fisher Scientific). RNA quality was evaluated by using a BioAnalyzer (Agilent Technologies, Santa Clara, CA, USA), and only samples with high-quality scores (RNA Integrity Number [RIN] ≥ 9) were used. RNAseq libraries were generated by using a TruSeq RNA sample preparation Kit v.2 (Illumina, San Diego, CA, USA) and sequenced by using a HiSeq1500 platform (Illumina, 100-bp single read, > 20 million mapped reads, Additional file 3: Table S2).

#### Adult tissues

Twenty-five target tissues were chosen from all around the adult body and dissected from a single adult individual to avoid overlapping sampling tissues (Fig. 3a). Four biological replicates (two males and two females of the d-rR strain) were prepared for each tissue. Dissected tissues were immediately homogenized in QIAzol reagent (Qiagen), and total RNA was isolated by using an RNeasy Mini kit (Qiagen) in accordance with the standard protocol. The quality and concentration of extracted RNA were evaluated in the same way as in the experiment done to extract RNA from embryos. For RNAseq library preparation, a TruSeq RNA sample preparation Kit v2 (Illumina) was used. The libraries were sequenced as paired-end (75-bp) reads on the NextSeq 550 platform (Illumina).

### Sex identification of embryos

After total RNA isolation, DNA–protein aggregates were precipitated in ethanol–sodium citrate solution (0.1 M sodium citrate in 10% ethanol), and DNA was further extracted from these aggregates by ethanol precipitation. By using the extracted DNA, the sex of embryos was determined by using the PCR-amplifying DMY gene, a Y-chromosome-specific gene known to initiate male sex differentiation after stage 34 [46] (forward primer sequence: 5′-TGTAGTCCAGAGGCTTCGTC-3′; reverse primer sequence: 5′-GGACGATGAAGCAGAGTAGC-3′).

### Estimation of gene expression levels from RNAseq data

Adapter trimming was performed by using the trimmomatic (ver. 0.38) program [47], and the quality of RNAseq data was evaluated by using FastQC (ver. 0.11.8, http://www.bioinformatics.babraham.ac.uk/projects/fastqc/). The sequenced reads were mapped against the reference medaka genome (version ASM223467v1) by using HISAT2 (ver. 2.1.0) [48]. The mitochondrial DNA sequence from the reference genome was removed before the mapping. StringTie (ver. 1.3.5, with all the default parameters) [49, 50] was used to quantify relative gene expression levels (in transcripts per kilobase million [TPM]). Random sub-sampling of 20 million reads from the total number of genome-mapped reads for each sample was performed to avoid bias arising from differences in read depth among samples. The log_10_-transformed TPM levels (namely, log_10_(TPM+1)) were used in the following analysis, where ${x}_j^i$ represents the log-transformed gene expression level of the *j*th gene in the *i*th individual. We then filtered out genes with low expression, keeping those that showed ${x}_j^i$ ≥ 0.1 for all individuals. Note that consistent results were obtained with other expression-level thresholds ranging from 0 to 1.5 (data not shown).

### Identification of genes with significantly greater deviation in expression levels from the technical error

In quantifying differences in phenotypes (whole embryonic transcriptome) and gene expression levels of same genes between individual embryos, bias from technical errors had to be reduced as much as possible. To do so, only genes showing significantly greater deviation in the inbred twin samples than in the technical replicates were used. (See also Additional file 1: Figure S5.) Notably, the deviations in expression of those genes expressed at low levels tended to be indistinguishable from the technical error. Technical replicates were prepared by pooling RNA from four individual embryos into one tube and then dividing the pooled sample into four subsamples. This was followed by RNAseq (Additional file 1: Figure S9). First, the difference in *j*th gene expression $\left|{x}_j^i-{x}_j^k\right|$ was calculated, where the *i*th and *k*th individuals are gender-matched twins. The technical error in *j*th gene expression was calculated as the average of $\left|{x}_j^{\mathrm{tech},i}-{x}_j^{\mathrm{tech},k}\right|$ over all possible combinations with *i* ≠ *k* (six combinations in total) among the four replicates, where ${x}_j^{\mathrm{tech},i}$ is the expression level of the *j*th gene in the *i*th technical replicate. Then, one-sided Wilcoxon rank-sum tests for each gene were performed to determine whether the inbred twins’ gene expression differences were significantly greater than those of the technical replicates (*α* = 0.01). Following the analysis, we used only those genes for which the expression differences were significantly larger than the technical error.

### Evaluation of whole embryonic developmental stability

The difference in whole embryonic gene expression profiles between inbred twins was quantified by calculating the variance of distribution of the differential gene expression levels. Let ${y}_j^{ik}$ be the difference in *j*th gene expression between the *i*th and *k*th individuals, i.e. ${y}_j^{ik}=\left({x}_j^i-{x}_j^k\right)$. Then, the variance was defined as ${V}^{ik}=\frac{1}{N}\sum_j{\left({y}_j^{ik}-\overline{y_j^{ik}}\right)}^2$, where $\overline{y^{ik}}$is the average gene expression difference across genes and *N* is the number of genes to be analysed. The developmental stability of the gene expression profiles was evaluated as the average of the variance *V*^*ik*^across gender-matched inbred twin pairs.

### Evaluation of read-depth bias toward whole embryonic variation

Potential bias from differences in read depth toward whole embryonic phenotypic variation was evaluated by creating a simulated dataset with different read depths. Genome-mapped RNAseq reads were randomly picked with several read depths (3-, 5-, 10-, 15-, 20-, 25-, and 30-million genome-mapped reads) and calculated the variance of distribution of differential gene expression (Additional file 1: Figure S3b). The test data set was obtained from the sequence data of gender-matched, quadruplet inbred twins (st. 23.5) raised in the same environment until sampling. Data for 20 million reads were created uniformly for all samples, as this was the maximum depth that could be obtained for all samples.

### Evaluation of whole embryonic evolutionary conservation

To quantify the intraspecies diversity of the embryonic gene expression profiles, the variance of the distribution of differential gene expression levels between two individuals were used, as defined above. After calculating *V*^*ik*^ for the *i*th Kasasa and *k*th Oura embryos among all possible gender-matched pairs and defined it as the intraspecies diversity. Intraspecies diversity was also calculated by using the same method as in previous studies by using 1 − Spearman’s correlation coefficient (rho) [7, 10, 12]. As shown in Additional file 1: Figure S4, consistent results were obtained between 1 − Spearman’s method and the analysis using the variance *V*^*ik*^. Note that adult fish of the Kasasa and Oura strains were kept in the same breeding environment.

### Evaluation of gene expression stability

The variation of each gene expression level was calculated by averaging the expression difference $\left|{x}_j^i-{x}_j^k\right|$ for all inbred twin pairs and defined as the gene expression stability of the *j*th gene. As the variation depends on the absolute expression level, we normalized it by using the following procedure (Additional file 1: Figure S9). For each developmental stage, (1) sort genes by the absolute expression levels averaged over all inbred individuals; (2) calculate a running median of the expression variation over the absolute expression levels (window size 501 genes). In other words, for each gene, the variation was corrected against those of ± 250 genes with similar expression levels; (2a) in the case of windows with fewer than 250 genes on either half side (e.g. the 250 genes with the top or bottom expression levels), reduce the window size to give an equal number of genes on each side; (3) obtain the corrected expression variation by subtracting the corresponding median value. This procedure eliminated the dependency of the expression variation on the absolute expression level (Additional file 1: Figure S9c).

### Evaluation of microevolutionary, intraspecies expression variation

To determine the intraspecies expression variation, we used the same calculation method that was used to evaluate the stability of gene expression. Namely, we calculated the expression difference between Kasasa and Oura embryos, $\left|{x}_j^{\mathrm{Kasasa},i}-{x}_j^{\mathrm{Oura},k}\right|$, over all gender-matched combinations, where ${x}_j^{\mathrm{Kasasa},i}$ and ${x}_j^{\mathrm{Oura},k}$ represent the expression level of the *j*th gene of the *i*th individual in the Kasasa population and the *k*th individual in the Oura population, respectively. We then normalized the intraspecies expression variation in the same way as for inbred twins to eliminate expression-level dependency.

### Evaluation of interspecies expression variation

Interspecies expression variation between medaka and other species was analysed as the expression difference between 1:1 orthologs (defined by reciprocal best BLAST hits using the longest peptide for each gene; e-value > 1e−5, BLAST+ ver. 2.9). The interspecies diversity between medaka and other species was also quantified as the difference in mean expression levels between 1:1 orthologs (defined by reciprocal best BLAST hits using the longest peptide for each gene; e-value > 1e−5, BLAST+ ver. 2.9). Given that there are no perfect counterparts in developmental stages between different species, we performed the analysis only for the most conserved developmental stage for each species, namely the phylotypic period (medaka, st. 23.5; zebrafish, prime-5; chicken, HH16; mouse, E9.0) [7, 10, 12, 28]. Peptide sequences of each species were obtained from the ensemble database (medaka, Ensembl v95/ASM223467v1; zebrafish, Ensembl v99/GRCz11; chicken, Ensembl v99/GRCg6a; mouse, Ensembl v99/GRCm38). For zebrafish, chicken, and mouse, the average expression level of each gene was calculated over biological replicates (3 for zebrafish, 2 for chicken, and 2 for mouse). For medaka, the average expression level was obtained over inbred individuals (46 for st. 15, 48 for st. 23.5, 50 for st. 28, and 26 for Hatch). The interspecies expression variation of the *j*th gene $\left|{x}_j^{\mathrm{medaka}}-{x}_j^{\mathrm{species}}\right|$ using species ∈ {zebrafish, chicken, mouse} was then calculated and normalized as mentioned above to eliminate expression-level dependency.

### Developmental genes and genes expressed through embryogenesis

#### Developmental genes

On the basis of gene-associated GO terms obtained from the Ensembl database (ver. 95), developmental genes were defined as those annotated with the GO term GO:0032502 [developmental process] or its descendant GO terms by using the GO.db package [51] (ver. 3.7.0) in R.

#### Genes expressed throughout embryogenesis

In accordance with previously published RNAseq data [39], genes were considered to be expressed throughout embryogenesis if they exhibited an average expression level among three biological replicates that was higher than the threshold (≥ 0.1) across all the sampled developmental stages [39] (16 developmental stages, Fig. 3c).

### GO slim enrichment analysis

GO slim terms for each gene were obtained by using the R package ‘biomaRt’ (ver. 2.38) [52, 53] with the Ensembl 95 medaka genome. After we had extracted the 10% of genes with the smallest variations, the GO slim terms of the remaining genes were analysed and an enrichment analysis was performed. One-sided Fisher’s exact tests were performed (false discovery rate ≤ 0.01, Benjamini–Hochberg procedure for multiple comparisons).

### Spatial and temporal pleiotropy of gene expression

Spatial pleiotropy of each gene was quantified as the number of tissues in which the gene was expressed among 25 tissues extracted from adult fish of the medaka d-rR strain (the strain of origin of the inbred Hd-rR strain). Genes with an average expression level TPM ≥ 1 among the replicate samples (two males and two females) were defined as expressed in that tissue. Temporal pleiotropy of each gene was quantified as the number of developmental stages in which the gene was expressed among the 16 developmental stages [39] (embryos of d-rR strain, three biological replicates for each stage). Genes with an average expression level TPM ≥ 1 were defined as being expressed at that stage.

### DNA extraction and genome resequencing

Genomic DNA for each medaka was extracted by using a DNeasy Blood and Tissue Kit (Qiagen) with RNase treatment. In the case of wild medaka strains (Kasasa and Oura strains from the same river system, and Tango and Kaishi strains from different river systems), one male individual from each strain was collected. Two male and two female twins were collected in the case of inbred medaka (Hd-rR). After we had evaluated DNA quality by using NanoDrop (Thermo Fisher Scientific), DNA libraries were prepared with a TruSeq DNA Nano LT Library Prep kit (Illumina, San Diego, CA, USA). Sequencing was performed by using a NextSeq 550 platform (Illumina, paired-end 150-bp reads).

### Evaluation of genomic diversity

Quality assessment and adapter trimming of DNAseq reads were performed in the same manner as for the RNAseq data. The sequence reads were aligned to the medaka reference genome (version, ASM223467v1) by using Bowtie2 (ver. 2.3.5.1) [54], and then only unique-hit reads were utilized. The aligned reads were further processed by using the ‘AddOrReplaceReadGroups’ command in Picard tools (ver. 2.20.8, http://broadinstitute.github.io/picard/) to assign read groups. Local realignment around indels was performed by using the Genome Analysis Toolkit [55] (GATK, ver. 3.8-1) ‘IndelRealigner’ command. For variant calling, the samtools [56] (ver. 1.9) ‘mpileup’ command was used, with default parameters. In the subsequent analysis, only high-quality sites [Phred-scaled quality score > 30, depth > ×5; filtered by using the bcftools [57] (ver. 1.9) ‘view’ command] were used. All the vcf files from different medaka samples were combined by using the ‘merge’ command in bcftools (ver. 1.9). PCA analysis was performed by using PLINK2.0 [58] (v2.00a2 64-bit, www.cog-genomics.org/plink/2.0/).

### Estimation of potential regulatory regions by using ATAC-seq data

Open chromatin regions were first identified by using previously published ATAC-Seq data [28] and were linked with the closest gene ID via the closest proximal transcription start site (TSS; done by using the ‘closest-features’ command implemented in BEDOPS, ver. 2.4.37) [59]. Potential regulatory regions were then defined as those that overlapped with the distal (±5000 bp from the TSS) and proximal (–100 to +50 bp from the TSS) regions. For the ATAC-seq signals located at the boundaries of these distal and proximal regions, only those that overlapped with more than half of the region were used. TSS information for each gene was obtained by using the R package ‘biomaRt’ (ver. 2.38) [52, 53] with the Ensembl 95 medaka genome.

### Single-nucleotide substitutions in open chromatin regions

The individual genomes of the following populations were mapped to the medaka reference genome (ver. ASM223467v1): Hd-rR (two males and two females), Kasasa (one male), Oura (one male), Kaishi (one male), and Tango (one male). Homozygous single-nucleotide substitutions from the reference genome were detected for sites with sufficient read quality and read depth (Phred-scaled quality score > 30, depth > ×5). Single-nucleotide substitutions within the open chromatin regions ±5000 bp around the TSS were assigned to each gene and counted across all eight samples.

### TATA-box elements

TATA-box elements in TSS proximal regions (−100 to +50 bp) were scanned by using the FIMO web browser [60] (ver. 5.3.3; *P* value cut-off 10^−4^; reference medaka genome, version ASM223467v1). The following four motif sequences were used to detect the TATA-box: TATAAAAA, TATAAATA, TATATAAA, and TATATATA. For ‘TATA’ repeated sequences, any two base shifts were counted separately. Those on different DNA strands were also counted separately.

### Statistics

Biological replicates consisted of embryos from different parents and born on different days to appropriately represent the population of interest. For statistical tests, *α* level = 0.05 was employed to indicate statistical significance throughout the analyses unless otherwise specified. To avoid an inflated type I error rate in multiple comparisons following the Kruskal–Wallis test, we performed a Steel–Dwass test implemented in the R package NSM3 (ver. 1.12) [61]. For GO slim term enrichment analysis, the Benjamini–Hochberg procedure was utilized with Fisher’s exact tests.

## Supplementary Information

**Additional file 1: Figure S1.** Geographical distribution and genetic diversity of Japanese medaka strains, as confirmed by genome resequencing. **Figure S2.** Quantification of phenotypic variation, its read-depth dependency and performance to classify different samples. **Figure S3.** Selecting genes with deviations significantly higher than technical errors. **Figure S4.** Whole embryonic phenotypic variations evaluated in various categories of gene sets. **Figure S5.** Expression-level differences of each gene in wild strains and inbred twins and correction for potential bias in gene expression variation. **Figure S6.** Representative developmental genes in the 10% of those with the highest or the lowest stability in gene expression levels. **Figure S7.** Genes with pleiotropic expression tend to have greater stability and higher conservation in microevolution. **Figure S8.** Features of the potential regulatory region did not significantly correlate with either gene expression stability or microevolutionary conservation. **Figure S9.** GO slim terms enriched in the 10% of genes with the least expression variation.

**Additional file 2: Table S1.** List of the genes with the least variation in gene expression levels for each stage.

**Additional file 3: Table S2.** Information on the all transcriptome data used in this study about RNAseq conditions, data quality, and repository ID.

## Data Availability

The datasets supporting the conclusions of this article are available in the DNA Data Bank of Japan repository through accession numbers DRA012427 [62] for the developmental transcriptome (experiment number DRX298419–DRX298634), DRA012432 [63] for tissue transcriptome (experiment number DRX298678–DRX298777), and DRA012429 [64] for genome resequencing (experiment number DRX298655–DRX298662). R scripts for the analyses in this paper are available on GitHub [65].

## Abbreviations

UV: Ultraviolet
LD50: Lethal dose 50
TPM: Transcripts per kilobase million
ATAC-seq: Assay for transposase accessible chromatin sequencing
GO: Gene Ontology
TSS: Transcription start site
