# Supplementary material for: Potential contribution of intrinsic developmental stability toward body plan conservation
Source: BMC Biol. 2022 Apr 11;20:82. doi: 10.1186/s12915-022-01276-5 (PMC8996622; doi:10.1186/s12915-022-01276-5)
Supplement: Supplementary file 1 — Additional file 1: Figure S1. Geographical distribution and genetic diversity of Japanese medaka strains, as confirmed by genome resequencing. Figure S2. Quantification of phenotypic variation, its read-depth dependency and performance to classify different samples. Figure S3. Selecting genes with deviations significantly higher than technical errors. Figure S4. Whole embryonic phenotypic variations evaluated in various categories of gene sets. Figure S5. Expression-level differences of each gene in wild strains and inbred twins and correction for potential bias in gene expression variation. Figure S6. Representative developmental genes in the 10% of those with the highest or the lowest stability in gene expression levels. Figure S7. Genes with pleiotropic expression tend to have greater stability and higher conservation in microevolution. Figure S8. Features of the potential regulatory region did not significantly correlate with either gene expression stability or microevolutionary conservation. Figure S9. GO slim terms enriched in the 10% of genes with the least expression variation. [file 12915_2022_1276_MOESM1_ESM.docx]

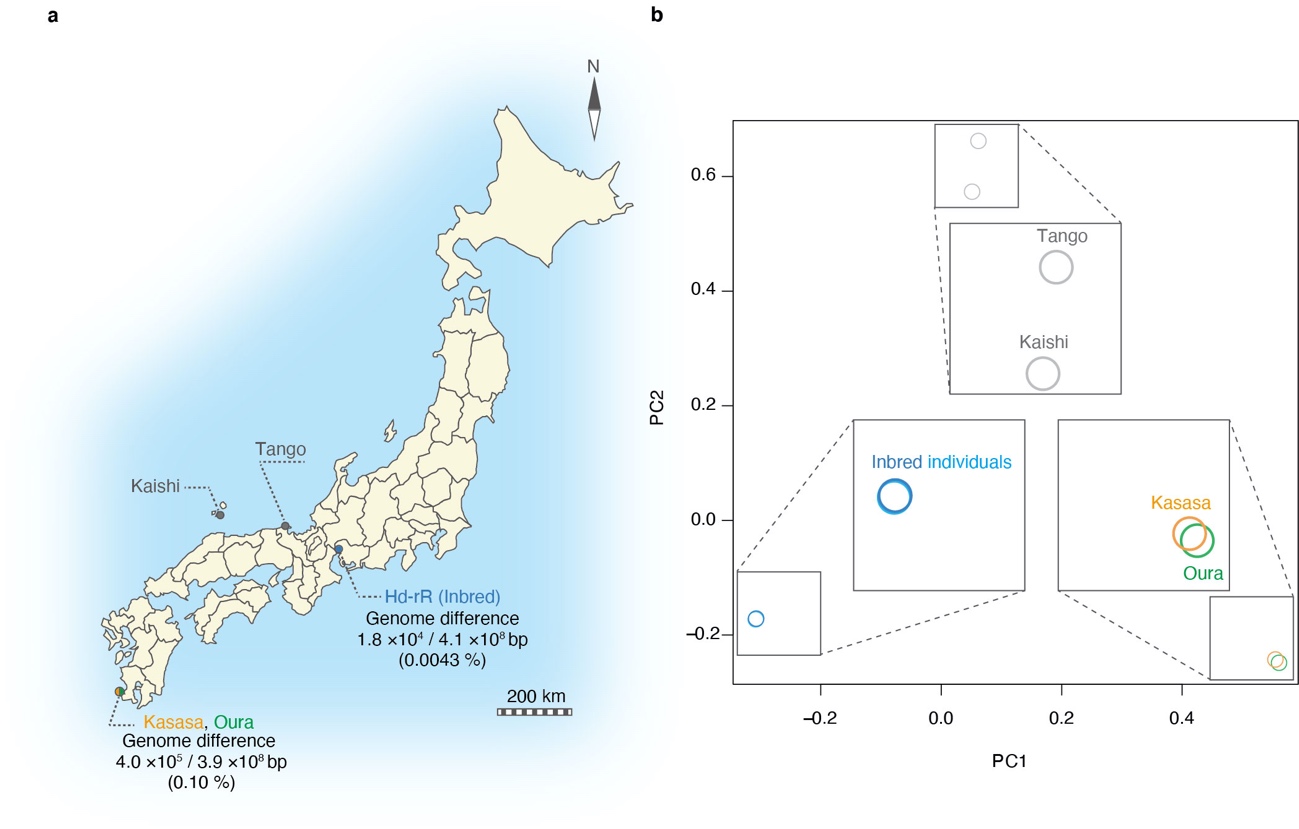


**Figure S1: Geographical distribution and genetic diversity of Japanese medaka strains, as confirmed by genome resequencing.**

**a.** Geographical distribution of the Japanese medaka strains used in this study. All these strains belonged to the medaka southern population[44]. Hd-rR was an inbred strain maintained at the National Institute for Basic Biology in Japan, whereas the others were wild strains. Kasasa and Oura strains lived in the same water system within 5 km of each other. Kaishi and Tango strains lived in different water systems about 270 km apart from each other. “Genome difference” indicates genetic diversity of the medaka strains, as estimated by whole genome resequencing of male individuals from each population. Only estimated homozygous sites in genome sequences with high read quality (Phred-scaled quality score > 30, depth > ×5) were selected for comparison to minimize bias from sequencing errors (denominator, see also **Methods**). The medaka reference genome is reported to be approximately 750 Mbp. Numerator is the number of sites where substitutions were found between individuals.**b.** Genomic diversities were visualized using principal component analysis. Note that the two inbred males (blue circles) are much closer to each other than the Kasasa–Oura males or Kaishi–Tango males.


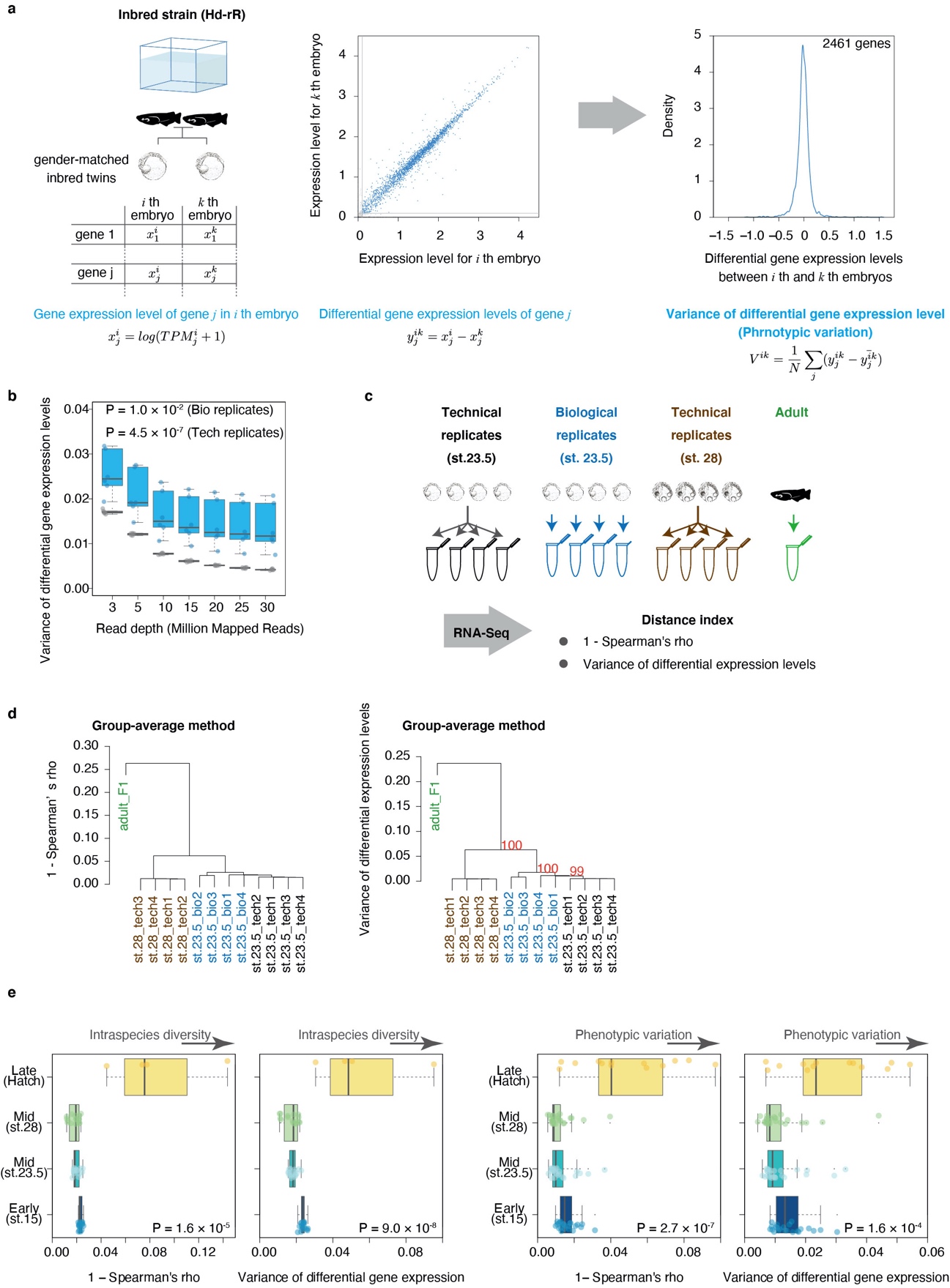


**Figure S2: Quantification of phenotypic variation, its read-depth dependency and performance to classify different samples.**

**a.** Whole embryonic phenotypic variations were estimated by calculating the variance of distribution of the differential gene expression levels between gender-matched, inbred twin embryos. (See also **Methods**.) Note that only genes that showed gene expression levels $x_{j}^{i}$ ≥ 0.1 in both twins were used for the analysis. Grey lines in the scatter plot indicate $x_{j}^{i}$ = 0.1 for each sample. **b.** The influence of sequence read depth on whole embryonic phenotypic variations. Two groups of data sets (technical replicates of st. 23.5 embryos, n = 4; biological replicates of st. 23.5 embryos, n = 4) were prepared. Datasets with different read depths (3 million to 30 million reads) were generated by random picking of genome-mapped reads. Box plots (blue) represent the phenotypic differences among biological replicates of st. 23.5 embryos, and box plots (grey) represent those among technical replicates. (**c-e**) Consistency between the variance and 1 – Spearman’s rho calculated to quantify the whole embryonic phenotypic variations. **c.** Four groups (technical replicates of st. 23.5 embryos, biological replicates of st. 23.5 embryos, technical replicates of st. 28 embryos, and an adult fish) of test datasets were prepared to determine to evaluate the distance indices. We expected that samples from technical replicates, from the same stage and from adult fish would be classifiable into different groups (see **Methods**). **d.** To test whether the four kinds of samples could be properly distinguished from each other, we performed a classification analysis. Phylogenetic trees were constructed by using the group-average method. Other clustering methods (single linkage, complete linkage and Ward’s methods) yielded consistent results (data not shown). **e.** Intraspecies diversity and phenotypic variation quantified by using the two indices showed consistent patterns. *P* values, Kruskal–Wallis test. Box plots: centre line, median; box limits, upper and lower quartiles; whiskers, 1.5× interquartile range; points, outliers.


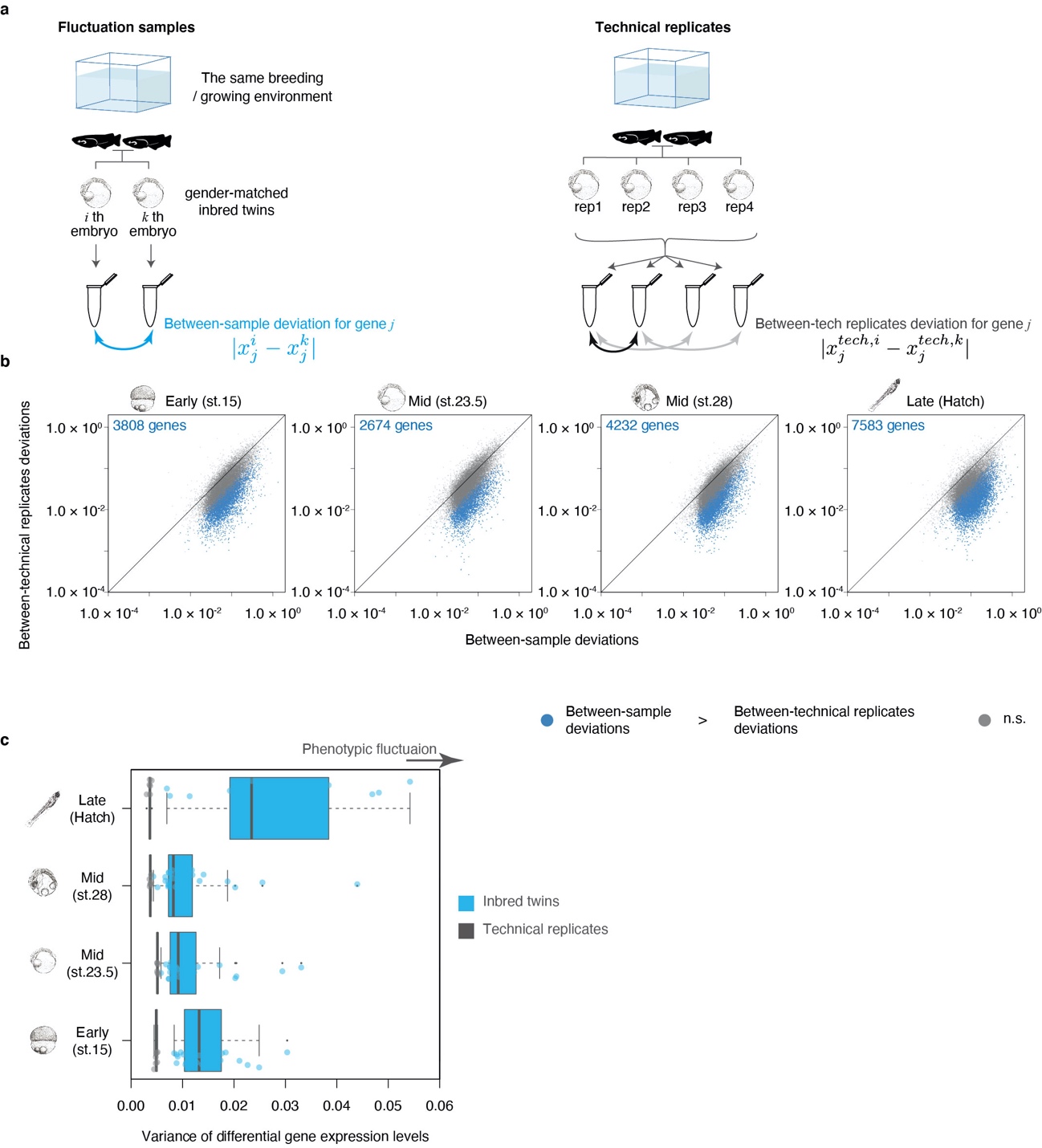


**Figure S3: Selecting genes with deviations significantly higher than technical errors.**

Only genes that showed significantly larger deviations in expression between inbred twins than in technical replicates were used to reduce the bias from technical errors. Technical error samples were prepared for each stage. **a.** Schematic illustrations of the process of calculation of gene expression deviations in inbred twins (left) and between technical replicates (right). Only genes with expression levels $x_{j}^{i}$ ≥ 0.1 in both embryos in a pair were used; otherwise the genes were treated as Not Available (N.A.) **b.** Genes with significantly larger (one-sided Wilcoxon rank-sum test, α-level = 0.01) deviations in expression between inbred twins than in technical replicates are coloured blue. These qualifying genes (numbers identified for each stage are shown at the top-left of each plot area) were used for the main analyses. **c.** Variances of distribution of differential gene expression levels in inbred twins and technical replicates are shown. Only the genes indicated in (b) were used for the analysis. Box plots: centre line, median; box limits, upper and lower quartiles; whiskers, 1.5× interquartile range; points, outliers.


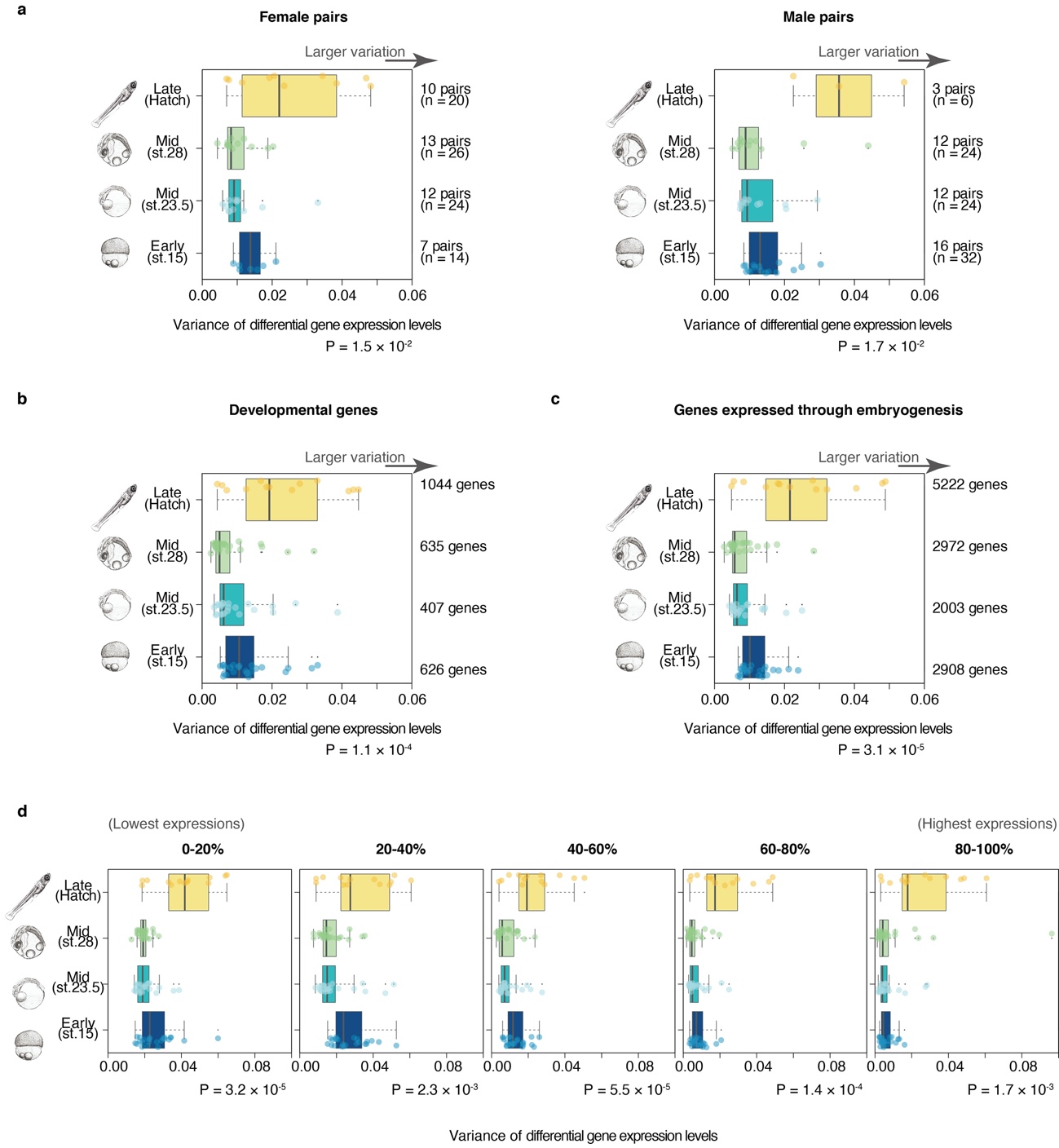


**Figure S4: Whole embryonic phenotypic variations evaluated in various categories of gene sets.**

The same analyses as in **Figure 1e** were performed on a sex-specific dataset (**a**), only with development-related genes (genes with the GO term GO:0032502 [developmental process] or its descendant terms (**b**), genes expressed in all 16 developmental stages (**c**), and genes categorized by relative expression levels (0% ≤ expression level < 20%, 20% ≤ expression level < 40%, 40% ≤ expression level < 60%, 60% ≤ expression level < 80%, 80% ≤ expression level ≤100% from the lowest expression level (**d**). Box plots: centre line, median; box limits, upper and lower quartiles; whiskers, 1.5× interquartile range; points, outliers.


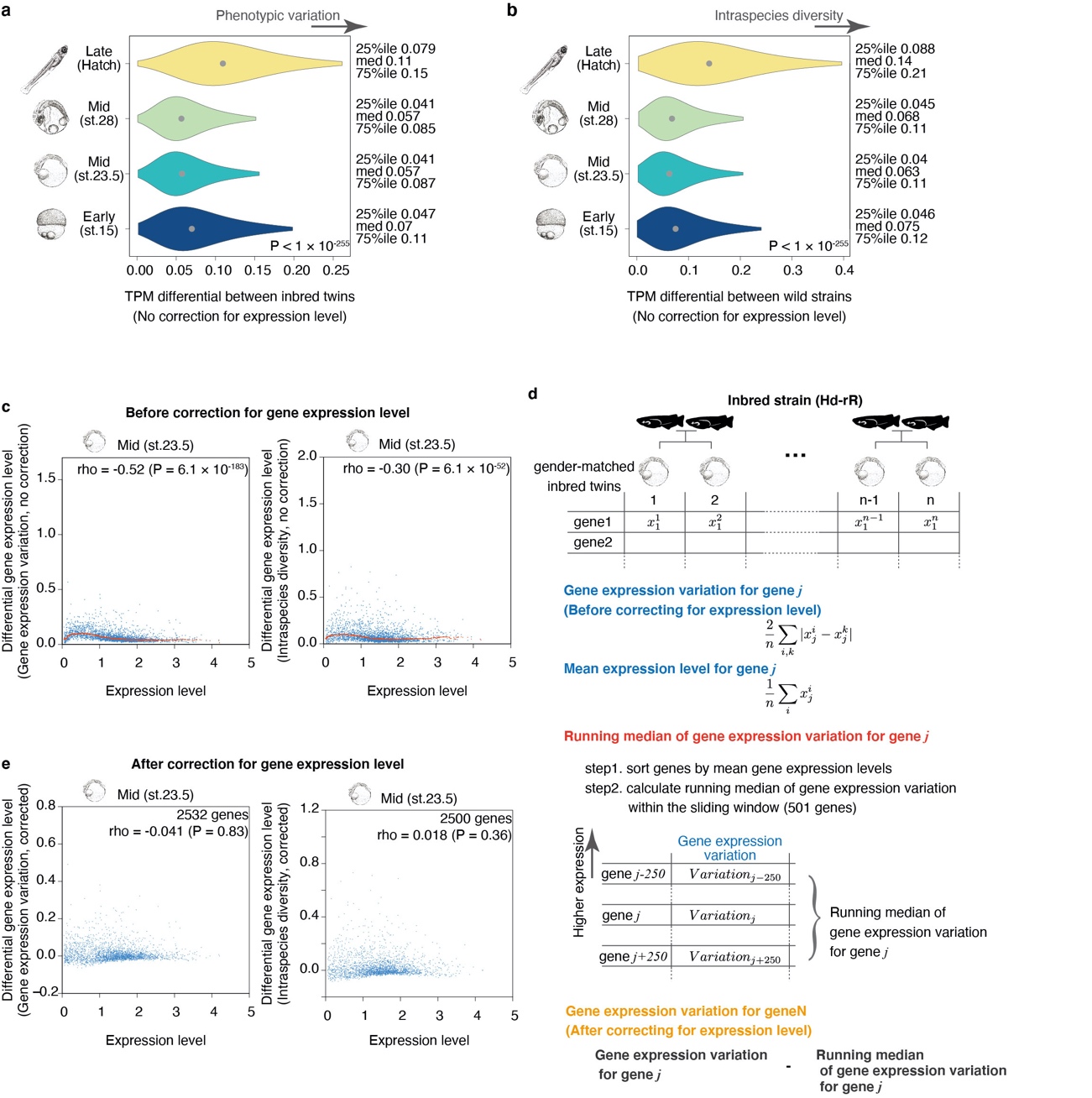


**Figure S5: Expression-level differences of each gene in wild strains and inbred twins and correction for potential bias in gene expression variation.**

(**a, b**) The absolute value of the expression level difference of each gene was calculated for gender-matched inbred twins (**a**, the estimated stability) and for wild individuals and visualized as violin plots (**b**, the estimated intraspecies diversity). A dot in the middle of the violin plot represents the median value for all the genes in the group. No correction for expression levels was performed in this analysis to match with the conditions used in **Figure 1**. P-value, Kruskal-Wallis test. (**c**) Relationships between gene expression level and differential expression level in inbred twins (left) and among embryos of wild populations (right) are shown as scatter plots (blue dots). Running median values of differential expression level (red dots) are overlaid. (**d**) Calculation method used to correct expression variation in relation to expression level. By using the average expression level of each gene (among all individuals at the same stage), genes were sorted and categorized by using a sliding window (size, 501 genes). Then, the median value of the expression variations among these 501 genes was calculated and subtracted from the variation value of the gene in the middle of the window. (See also **Methods.**) (**e**) Differential gene expression levels in inbred twins (left) and among embryos of wild populations (right) were corrected for bias from their expression levels by using the running median values; the relationships between gene expression levels and the corrected differential expression levels are shown. Spearman’s correlation coefficients are shown in each plot. *P* values, test of no correlation.


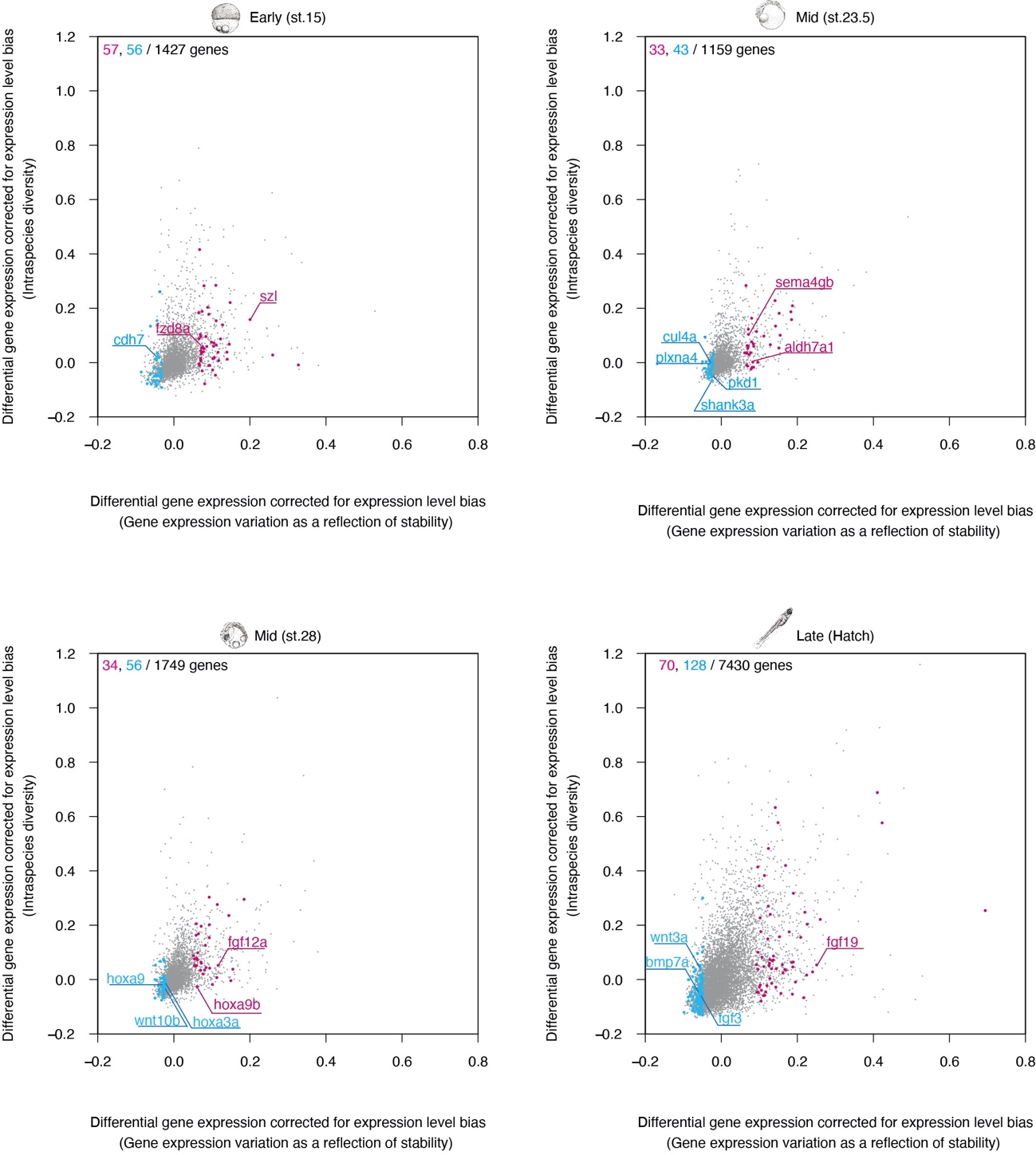


**Figure S6: Representative developmental genes in the 10% of those with the highest or the lowest stability in gene expression levels.**

Relationships between gene expression variation in inbred twins (x-axis, stability in gene expression levels) and intraspecies diversity (y-axis), as measured between the Kasasa and Oura strains. Blue dots are the 10% of developmental genes (genes with the GO term GO:0032502 [developmental process] or its descendant terms) with the least variation in gene expression. Pink dots are the 10% of developmental genes with the greatest variation in gene expression. Grey dots are other, background, genes. Names of some representative developmental genes are shown. The names of all the genes with the least variation in gene expression can be found in Additional file 2: **Table S1**.


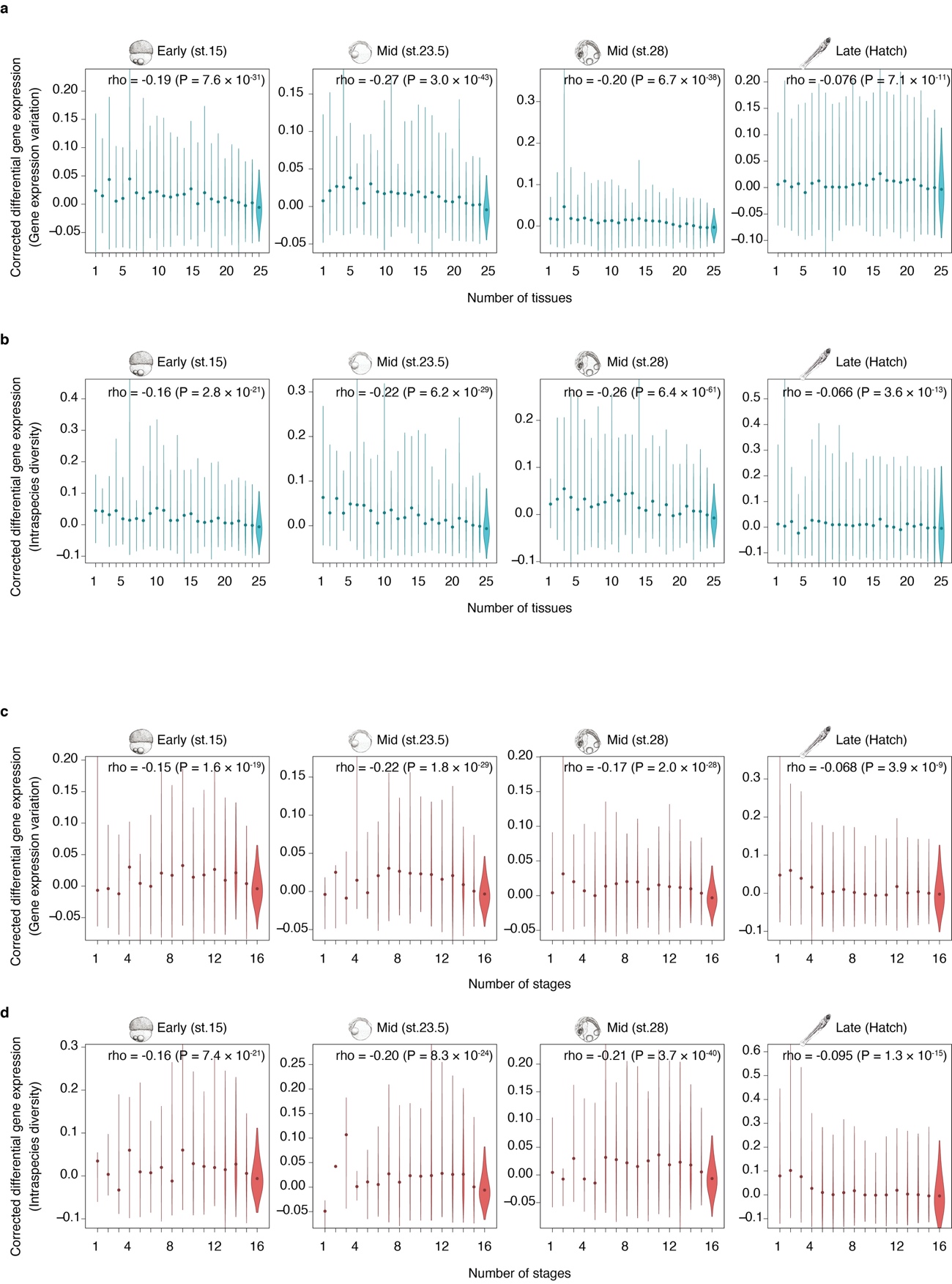


**Figure S7: Genes with pleiotropic expression tend to have greater stability and higher conservation in microevolution.**

**a.** Relationship between spatial pleiotropy, as estimated from an analysis of 25 adult tissues, and expression variation (as a reflection of gene expression stability), as estimated in inbred twins. Violin plots are shown for each gene set categorised by the number of tissues in which the genes were expressed. x-axis shows the number of adult tissues in which the genes were expressed (mean TPM ≥ 1 among the four replicates). y-axis: gene expression variations (gene expression stability) estimated from inbred twins. **b.** Relationship between spatial pleiotropy and intraspecies diversity (differential expression levels), as estimated in the Kasasa vs. Oura populations. x-axis shows the number of adult tissues in which the genes were expressed (mean TPM ≥ 1 among the four replicates). y-axis shows intraspecies diversity. **c.** Relationship between temporal pleiotropy (as estimated by using 16 developmental stages) and gene expression variation, as estimated in inbred twins. x-axis shows the number of developmental stages in which the genes were expressed (mean TPM ≥ 1 among the three replicates). y-axis shows gene expression variation (gene expression stability), as estimated in inbred twins. **d.** Relationship between temporal pleiotropy and intraspecies diversity. Genes detected in each stage were categorized by the numbers of developmental stages in which they were expressed (mean TPM ≥ 1 among the three replicates); their intraspecies diversity, as estimated in the Kasasa vs. Oura populations, is shown as violin plots. Spearman’s correlation coefficient and *P* values (test of no correlation) are shown in each plot. Violin plots represent genes within the 1.5× interquartile range, and width of violin plot areas reflects the gene numbers. Dots in the middle of violin plots represent median values. The analyses were done for gene expression variations and diversity in each developmental stage.


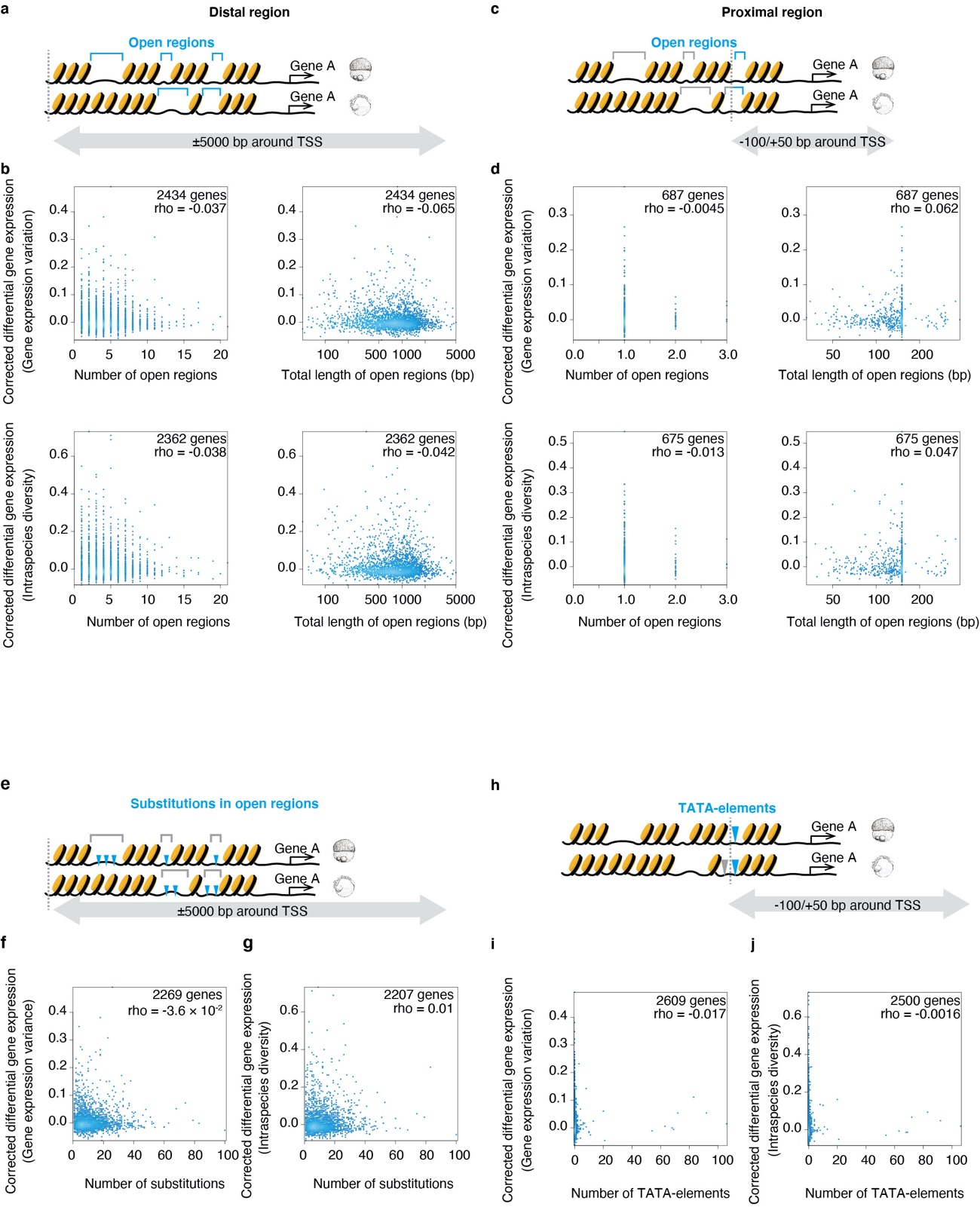


**Figure S8: Features of the potential regulatory region did not significantly correlated with either gene expression stability or microevolutionary conservation.**

Open chromatin regions within ±5000 bp (distal, **a, b, e-g)** or –100 to +50 bp (proximal, **c, d, h-j**) of the transcription start site (TSS) were identified by using previously published ATAC-Seq data[28] and were defined as potential regulatory regions for each gene. **b, d.** The relationship between the number of open chromatin regions (left panels) or the total length of potential regulatory regions (right panels) and gene expression variation as an indicator of expression stability (top panels) or intraspecies diversity (bottom panels) is shown. **f, g.** The relationships between the number of estimated homozygous single nucleotide substitutions within open chromatin regions and gene expression variation (gene expression stability, **f**) or intraspecies diversity (**g**) is shown. The number of estimated homozygous single nucleotide substitutions within open chromatin regions was summed over all genome-resequenced inbred and wild individuals. (See also **Methods**.) **i, j.** The relationship between the number of TATA-box motifs (‘TATAWAWA’ sequence) in the core promoter region (–100 to + 50 bp from TSS) and gene expression stability (**i**) or intraspecies diversity (**j**) is shown. If the ‘TATA’ sequence was repeated, any two base shifts were counted separately. Gene expression variation (gene expression stability) was estimated in inbred twins, whereas intraspecies diversity was estimated in embryos from the Kasasa and Oura populations. The results here represent data (ATAC-Seq, diversity and stability) obtained at st. 23.5. Similar trends were obtained in the other stages (data not shown). Spearman’s correlation coefficient and *P* values (test of no correlation) are shown in each plot.


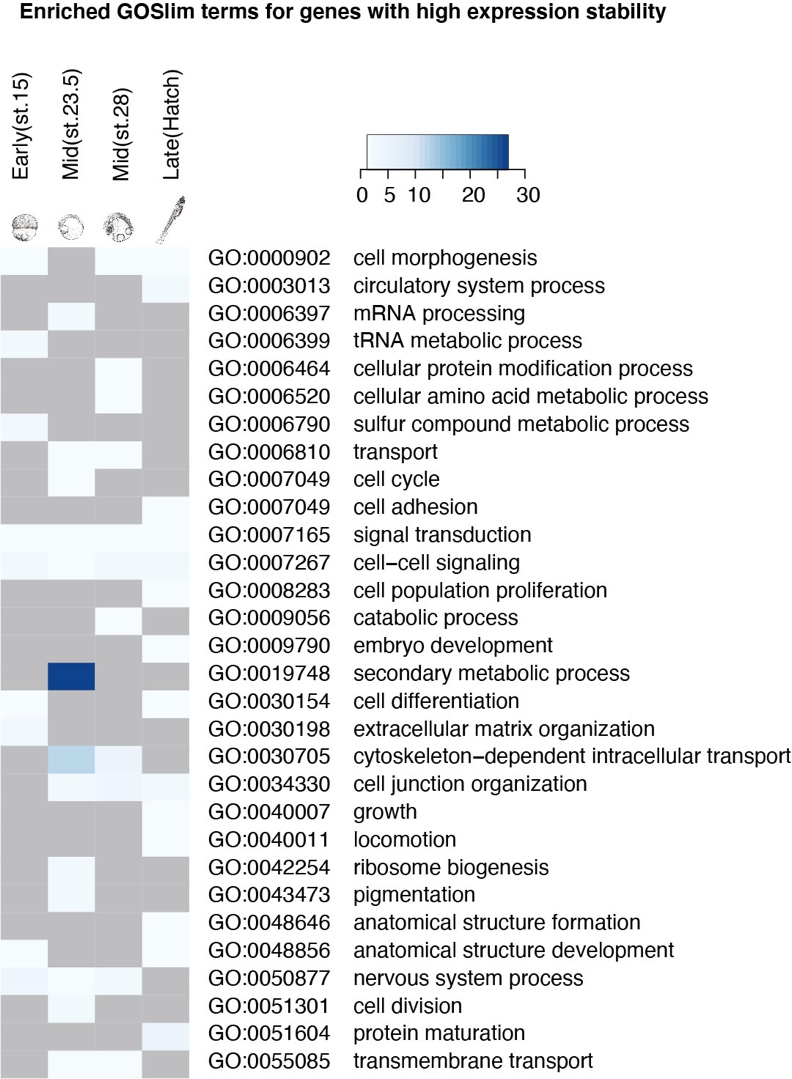


**Figure S9: GO slim terms enriched in the 10% of genes with the least expression variation.**

Shown are GO slim terms enriched in the 10% of genes with the least expression variation compared with those in the remaining 90% of genes. Colour scale represents the ratio between the expected value and the observed number of terms, that is {(number of GO term counts in 10% of genes / number of total GO term counts in 10% of genes) / (number of GO term counts in 90% of genes / number of total GO term counts in 90% of genes)}. Only GO slim terms with false discovery rate (FDR) ≤ 0.01 (Benjamini–Hochberg procedure) are shown; those with FDR > 0.01 are greyed out.
